# Protein tyrosine phosphatase Shp2 deficiency in podocytes attenuates lipopolysaccharide-induced proteinuria

**DOI:** 10.1038/s41598-017-00564-3

**Published:** 2017-03-28

**Authors:** Ming-Fo Hsu, Ahmed Bettaieb, Yoshihiro Ito, James Graham, Peter J. Havel, Fawaz G. Haj

**Affiliations:** 1 Department of Nutrition, University of California Davis, One Shields Ave, Davis, CA 95616 USA; 2 Department of Molecular Biosciences, School of Veterinary Medicine, University of California Davis, One Shields Ave, Davis, CA 95616 USA; 3 Comprehensive Cancer Center, University of California Davis, Sacramento, CA 95817 USA; 4 Division of Endocrinology, Diabetes, and Metabolism, Department of Internal Medicine, University of California Davis, Sacramento, CA 95817 USA; 5 Department of Nutrition, University of Tennessee-Knoxville, Knoxville, TN 37996 USA

## Abstract

Podocytes are specialized epithelial cells that play a significant role in maintaining the integrity of the glomerular filtration barrier and preventing urinary protein leakage. We investigated the contribution of protein tyrosine phosphatase Shp2 to lipopolysaccharide (LPS)-induced renal injury. We report increased Shp2 expression in murine kidneys and cultured podocytes following an LPS challenge. To determine the role of podocyte Shp2 *in vivo*, we generated podocyte-specific Shp2 knockout (pod-Shp2 KO) mice. Following administration of LPS, pod-Shp2 KO mice exhibited lower proteinuria and blood urea nitrogen concentrations than controls indicative of preserved filter integrity. In addition, renal mRNA and serum concentrations of inflammatory cytokines IL-1β, TNFα, INFγ and IL-12 p70 were significantly decreased in LPS-treated knockout mice compared with controls. Moreover, the protective effects of podocyte Shp2 deficiency were associated with decreased LPS-induced NF-κB and MAPK activation, nephrin phosphorylation and attenuated endoplasmic reticulum stress. These effects were recapitulated in differentiated E11 murine podocytes with lentiviral-mediated Shp2 knockdown. Furthermore, Shp2 deficient podocytes displayed reduced LPS-induced migration in a wound healing assay. These findings identify Shp2 in podocytes as a significant contributor to the signaling events following LPS challenge and suggest that inhibition of Shp2 in podocytes may present a potential therapeutic target for podocytopathies.

## Introduction

The glomerulus is the basic filtration unit of the kidney that prevents macro-molecules from being lost into the urine, and an effective glomerular filtration barrier (GFB) is critical for normal renal function^1^. Structurally the GFB is composed of podocytes, glomerular basement membrane, and endothelium. Podocytes are differentiated cells with extended membrane branches termed foot processes that interdigitate together to form unique intercellular junctions called slit diaphragms. Foot processes flattening damages filtration barrier integrity and correlates with development of proteinuria in humans and animal models of podocyte injury^2^. Proteinuria is an early marker of renal injury and may accelerate disease progression towards renal failure^3^. Therefore, understanding the intricate signaling mechanisms underlying podocyte dysfunction is essential for developing effective therapies for proteinuria.

Protein tyrosine phosphorylation and dephosphorylation are fundamental signaling mechanisms that contribute to both normal podocyte function and repair after injury^4^. Changes in phosphotyrosine-based signaling are mediated by the dynamic and opposing actions of protein tyrosine kinases and protein tyrosine phosphatases (PTPs)^5^. A growing body of evidence suggests a role for PTPs in podocyte function and repair. Biochemical studies *in vitro* and rodent models implicate protein tyrosine phosphatase 1B (PTP1B) in podocyte function. PTP1B inhibition attenuates complement-mediated glomerular injury^6^ and protects against podocyte injury and proteinuria^7^. Also, PTP1B can regulate podocyte function through tyrosine phosphorylation of nephrin, a key podocyte protein that regulates actin cytoskeleton^8^. Moreover, expression of the Src homology 2 (SH2) domain-containing phosphatase 1 (Shp1) increases in podocytes after hyperglycemia leading to decreased nephrin tyrosine phosphorylation^9^. Together, these studies establish a role for some PTPs in podocyte function, but additional investigation into the contribution of these enzymes to podocytopathies is warranted.

Src homology 2 domain-containing phosphatase 2 (Shp2; encoded by *PTPN11*) is a ubiquitously expressed non-transmembrane phosphatase. Shp2 contains two N-terminal SH2 domains, a tyrosine phosphatase domain, and a C-terminal region with phosphorylation sites^10^. The SH2 domains target Shp2 to specific phosphotyrosine proteins, including some receptor tyrosine kinases (RTKs) and several scaffolding adaptors^11^. Shp2 plays an essential role in most RTK signaling pathways, where it is required for normal activation of the extracellular signal-regulated kinase (ERK) pathway, and its downstream transcriptional targets^12, 13^. Accordingly, Shp2 plays a significant role in maintaining homeostasis by regulating many facets of cell signaling. Recent studies establish an association of Shp2 with nephrin and demonstrate that podocytes lacking Shp2 do not develop foot process spreading after injury *in vivo* using protamine sulfate or nephrotoxic serum models^14^. In this study, we determined the contribution of Shp2 in podocytes to LPS-induced podocyte injury and investigated the underlying mechanisms.

## Results

### Increased renal and podocyte Shp2 expression upon LPS treatment

**Figure 1:**
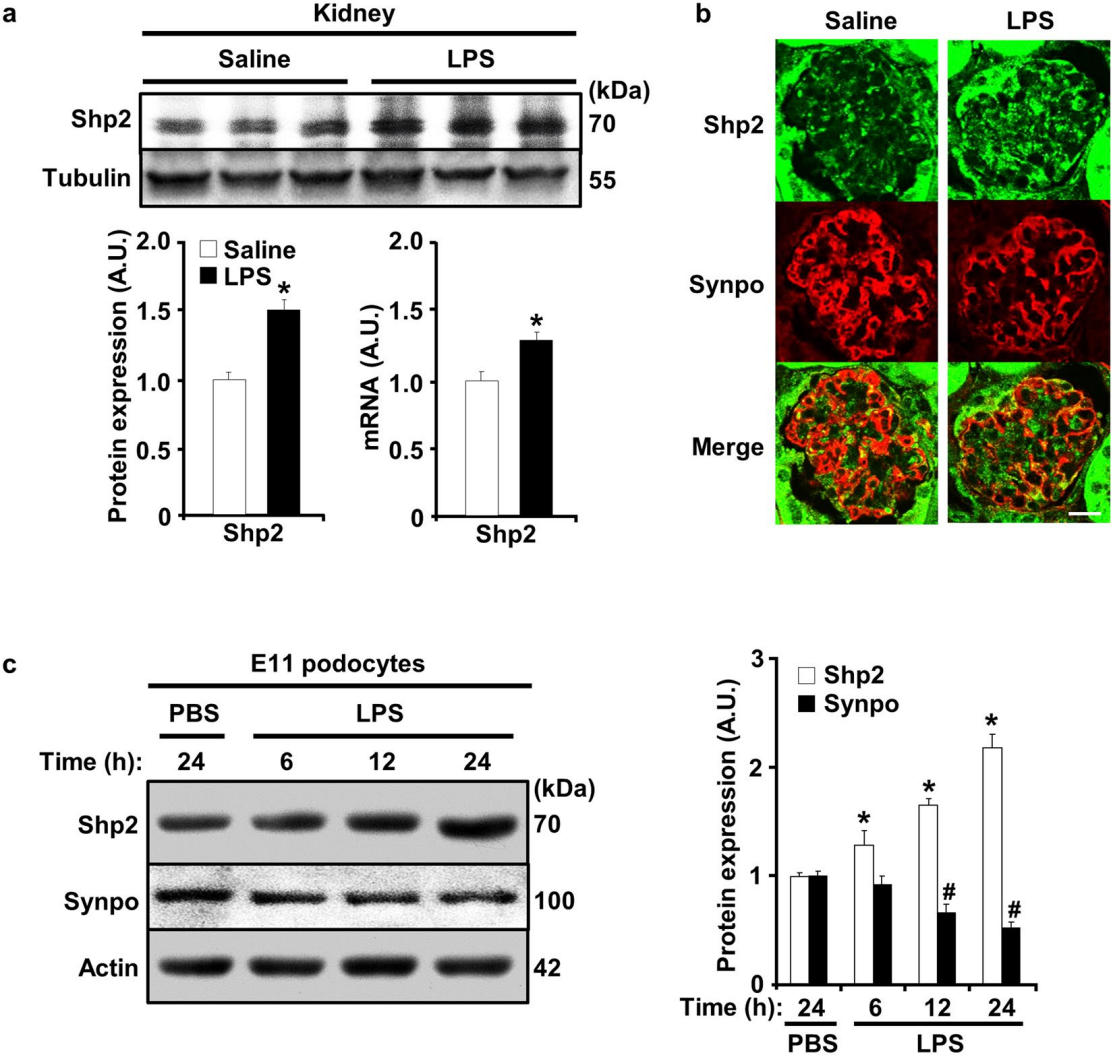
LPS treatment increases renal and podocyte Shp2 expression. (**a**) Immunoblots of Shp2 and tubulin in total kidney lysates. Control (saline, n = 6) and LPS-injected (n = 6) wild-type male mice were sacrificed 24 hours after injection. Representative images are shown and each lane represents an animal. Shp2 protein expression (left, normalized to tubulin) and mRNA (right, normalized to *Tbp*) presented in bar charts as means + SEM. *p < 0.05 indicates a significant difference between saline and LPS-treated mice. (**b**) Co-immunostaining of Shp2 (green) and synaptopodin (Synpo, red) in kidney sections from saline and LPS-treated wild-type mice. Scale bar: 20 µm. (**c**) Differentiated E11 podocytes were treated with PBS for 24 hours and with LPS for 6, 12 and 24 hours. Cell lysates were immunoblotted for Shp2, synaptopodin, and actin. Protein expression was normalized to actin and presented in a bar chart as means + SEM from three independent experiments. *p < 0.05 and ^#^ *p* < 0.05 indicate a significant difference between PBS (24 h) and LPS-treated groups for Shp2 and synaptopodin, respectively. A.U., arbitrary units.

Shp2 is differentially expressed in various types of renal tumors^15^, but its expression in normal kidneys and podocytes remains largely unexplored. Recently, Verma *et al*. report increased Shp2 phosphorylation at Tyr542, a putative marker of enzyme activity, in a subset of human glomerular diseases^14^. We investigated renal Shp2 transcript and protein expression in LPS-treated wild-type mice as detailed in methods. Immunoblots of total kidney lysates revealed a significant increase in Shp2 at both transcript and protein levels upon LPS treatment (Fig. 1a). Similarly, co-immunostaining of Shp2 and synaptopodin demonstrated LPS-induced increase in Shp2 expression in podocytes (Fig. 1b). Also, Shp2 expression was determined in differentiated E11 murine podocytes treated with LPS for 6, 12 and 24 hours. Immunoblotting revealed a time-dependent, LPS-induced increase in Shp2 protein expression, concomitant with decreased synaptopodin expression as previously reported^16^ (Fig. 1c). Thus, Shp2 expression is modulated in kidneys and podocytes upon LPS challenge.

### Mice with podocyte Shp2 deficiency are resistant to LPS-induced injury

**Figure 2:**
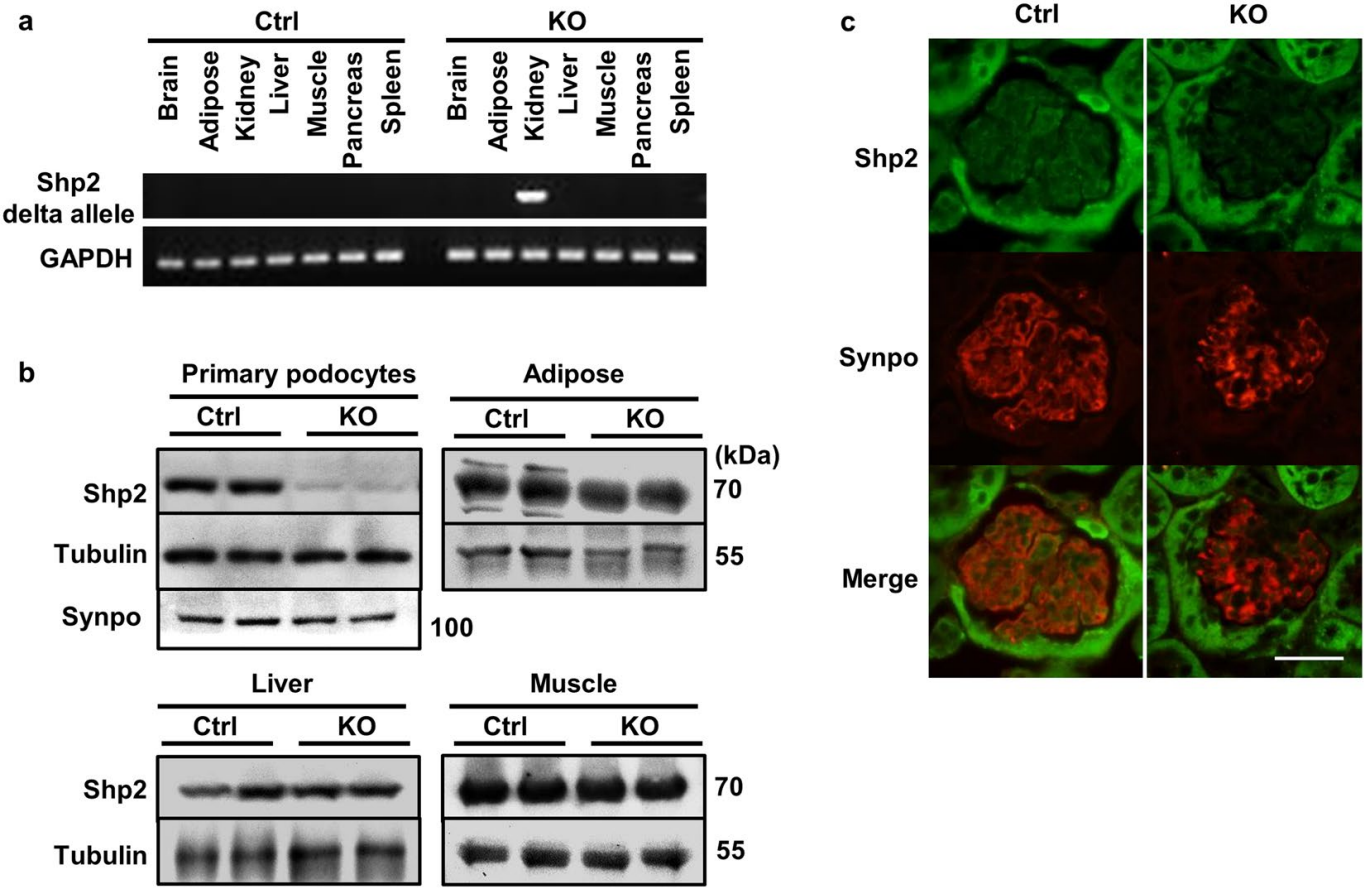
Efficient and specific deletion of Shp2 in podocytes. (**a**) Genomic DNA was extracted from tissues (as indicated) of control (Ctrl) and pod-Shp2 knockout (KO) mice. Deletion of the floxed allele was detected by PCR, and GAPDH served as a loading control. (**b**) Immunoblots of Shp2 protein expression in isolated primary podocytes, adipose (epididymal fat), liver and muscle from Ctrl and KO mice. Tubulin and synaptopodin (Synpo, for podocytes) presented as loading controls. (**c**) Immunostaining of Shp2 (green) and synaptopodin (red) in kidney sections from Ctrl and KO mice. Scale bar: 50 µm.

Upregulation of renal Shp2 expression upon LPS challenge led us to hypothesize that Shp2 may play a role in signaling events following LPS-induced podocyte injury. To that end, we generated mice with podocyte-specific Shp2 deletion as detailed in methods. Podocyte Shp2 knockout mice (hereafter termed pod-Shp2 KO) appeared healthy and with no gross defects in the kidneys. Deletion of Shp2 was confirmed using PCR, biochemical and immuno-histochemical approaches. The recombined Shp2 product was detected only in kidneys of pod-Shp2 KO mice (Fig. 2a). In addition, immunoblots of primary podocytes from control and pod-Shp2 KO mice demonstrated ablation of Shp2 in knockout mice (Fig. 2b). Shp2 expression was comparable in other tissues (adipose, liver, and muscle) of control and knockout mice suggesting specificity of deletion. Moreover, immunostaining of Shp2 and synaptopodin in kidney sections of control and pod-Shp2 KO mice further demonstrated deletion of Shp2 in podocytes of knockout mice (Fig. 2c). Collectively, these data demonstrate efficient and specific deletion of Shp2 in podocytes and establish pod-Shp2 KO mice as a suitable model to investigate the potential contribution of Shp2 to LPS-induced renal injury.

**Figure 3:**
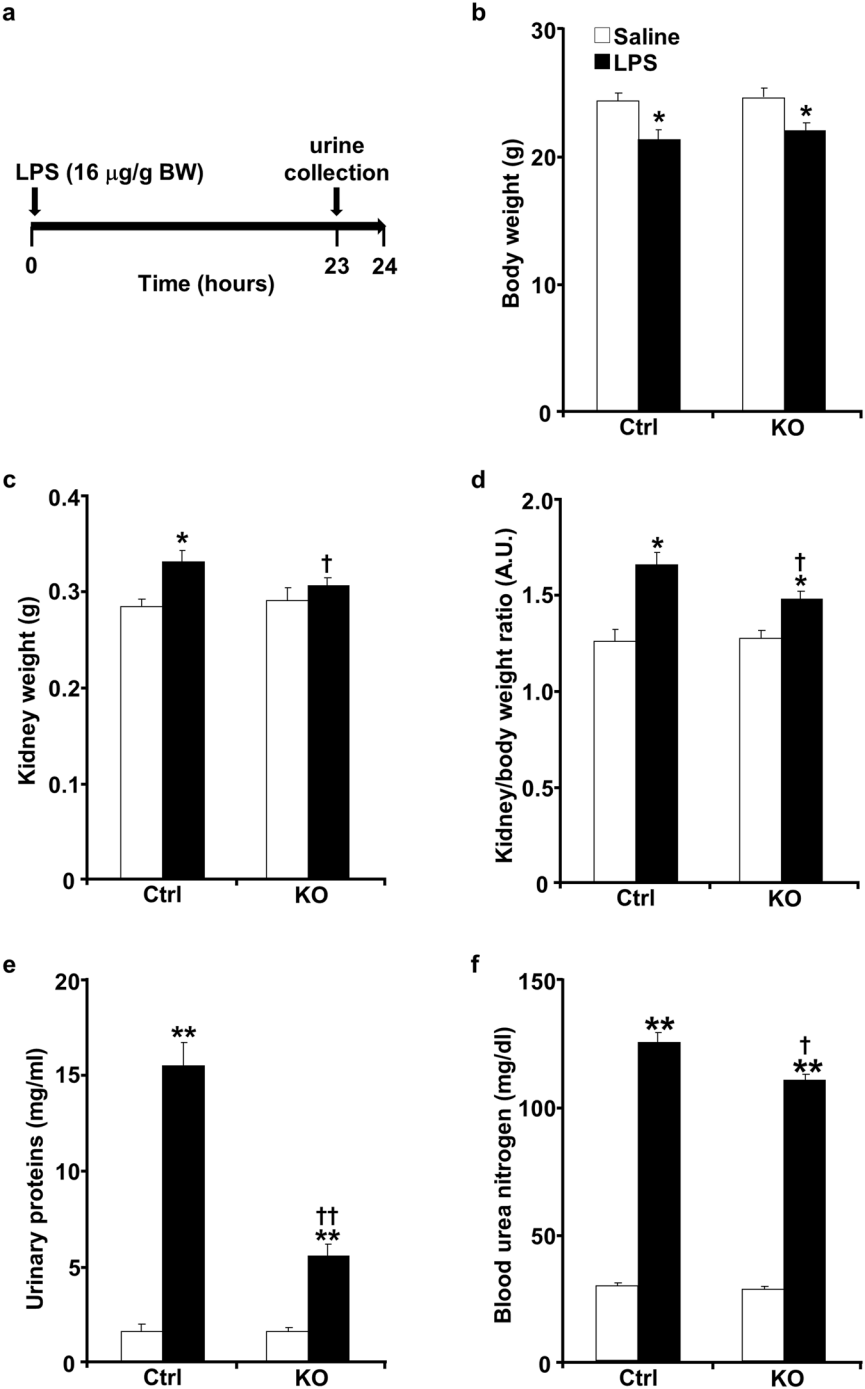
Pod-Shp2 KO mice are more resistant than controls to LPS-induced renal injury. (**a**) Schematic of experimental timeline for LPS administration and mice sacrifice. Body weight (**b**), kidney weight (**c**), kidney/body weight ratio (**d**), urinary proteins concentration (**e**) and blood urea nitrogen (**f**) of control (Ctrl, n = 8) and pod-Shp2 knockout (KO, n = 8) mice without (saline) and with LPS treatment. *p < 0.05 and **p < 0.01 indicate a significant difference between saline and LPS treatments; ^†^ *p* < 0.05 and ^††^ *p* < 0.01 indicate a significant difference between Ctrl and KO mice. Data were presented as means + SEM. A.U., arbitrary units.

To evaluate the effects of podocyte Shp2 deficiency on renal injury, age-matched control, and pod-Shp2 KO male mice were treated with LPS and sacrificed after 24 hours (Fig. 3a). LPS led to a comparable mild reduction in body weight of control and knockout mice (Fig. 3b). However, LPS-induced increase in kidney weights was significantly higher in control compared with knockout mice (Fig. 3c,d). Notably, LPS induced a significant increase in total urinary proteins, but that was lower in knockout mice than controls indicative of preserved filter integrity and renal function (Fig. 3e). Consistent with these observations, LPS-induced increase in blood urea nitrogen was significantly lower in knockout mice compared with controls (Fig. 3f). Similar findings were obtained using female mice establishing that the effects of podocyte Shp2 deletion on the LPS-induced injury were not gender specific (Fig. S1). Moreover, the nephrotoxic nephritis model of renal injury also demonstrated protective effects of podocyte Shp2 deficiency as evidenced by lower urine albumin/creatinine and blood urea nitrogen in knockout mice than controls (Fig. S2). Together, these data establish protective effects of podocyte Shp2 deficiency against LPS-induced renal dysfunction.

### Decreased systemic and renal inflammation in pod-Shp2 KO mice after LPS challenge

**Figure 4:**
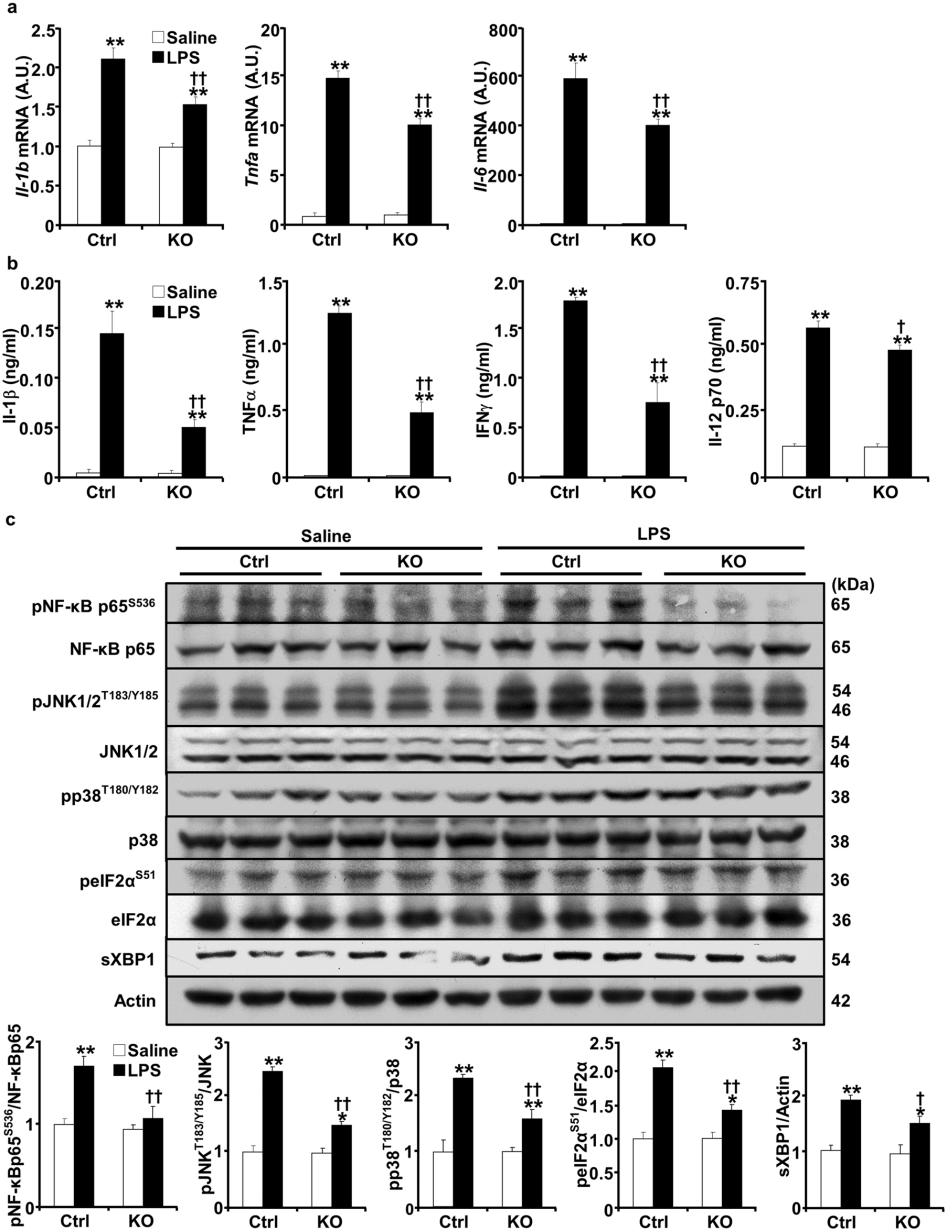
Attenuated LPS-induced inflammatory response in pod-Shp2 KO mice. Renal mRNA of *Il*-*1b*, *Il*-*6* and *Tnfa* (**a**), and plasma concentrations of IL-1β, TNFα, INFγ and IL-12 p70 (**b**) in saline and LPS-treated control (Ctrl, n = 6) and pod-Shp2 knockout (KO, n = 6) mice. (**c**) Kidney lysates from Ctrl and KO mice without (saline) and with LPS treatment were immunoblotted for phosphorylated NF-κB p65, JNK, p38, peIF2α and their respective proteins, spliced XBP1 (sXBP1), and actin as a loading control. Representative images are shown. Bar charts represent pNF-κB p65/NF-κB p65, pJNK/JNK, pp38/p38, peIF2α/eIF2α and sXBP1/actin as means + SEM. For all bar charts, *p < 0.05 and **p < 0.01 indicate a significant difference between saline and LPS treatments; ^†^ *p* < 0.05 and ^††^ *p* < 0.01 indicate a significant difference between Ctrl and KO mice. A.U., arbitrary units.

LPS treatment increases systemic and renal inflammatory cytokines in rodent models^17, 18^. We evaluated the effects of podocyte Shp2 deficiency on LPS-induced circulating and renal inflammatory cytokines. Consistent with published reports^17, 19^, interleukin-1b (*Il*-*1b*), interleukin-6 (*Il*-*6*) and tumor necrosis factor alpha (*Tnfa*) renal mRNA, as well as IL-1β, TNFα, interferon-γ (INFγ) and interleukin-12 (IL-12) p70 serum concentrations were significantly increased in LPS-treated mice (Fig. 4a,b). However, LPS-induced increase of inflammatory cytokines was significantly less in pod-Shp2 KO mice compared with controls. Similar attenuation of inflammatory markers was also observed in female knockout mice (Fig. S3).

**Figure 5:**
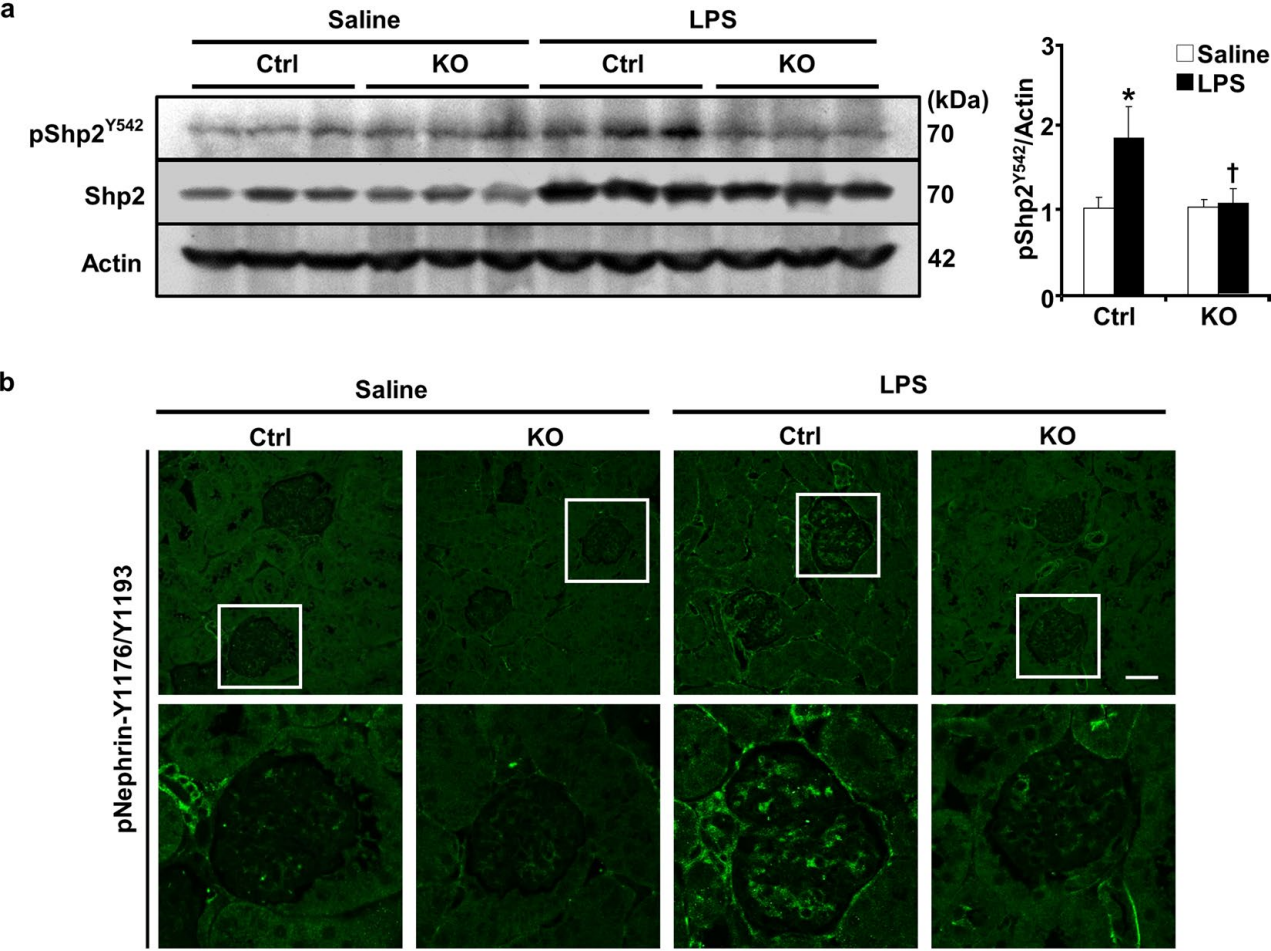
Attenuated LPS-induced Shp2 and nephrin phosphorylation in pod-Shp2 KO mice. (**a**) Kidney lysates from control (Ctrl) and pod-Shp2 knockout (KO) mice without (saline) and with LPS treatment were immunoblotted for pShp2 (Tyr542), Shp2 and actin as a loading control. Representative images are shown and each lane represents an animal. Bar chart represents pShp2 (Tyr542)/actin as means + SEM (n = 6). *p < 0.05 indicates a significant difference between saline and LPS treatments; ^†^ *p* < 0.05 indicates a significant difference between Ctrl and KO mice. A.U., arbitrary units. (**b**) Immunostaining of pNephrin (Y1176/Y1193) in kidney sections from Ctrl and KO mice without (saline) and with LPS treatment. Lower panel includes enlarged images that are highlighted by white boxes in the upper panel. Scale bar: 50 µm.

LPS is primarily recognized by toll-like receptor 4 (TLR4)^20, 21^. Through TLR4 activation the nuclear factor-kappa B (NF-κB) and MAPK (such as JNK and p38) pathways are significant effectors for cytokine production and LPS-induced inflammation^22^. Accordingly, activation of NF-κB, JNK and p38 was determined in kidneys of control and knockout mice under basal and LPS-treated conditions. LPS treatment induced a significant increase in NF-κB, JNK and p38 phosphorylation in control mice but that was significantly less in pod-Shp2 KO mice (Fig. 4c). In addition, endoplasmic reticulum (ER) stress is implicated in the pathogenesis of proteinuric kidney diseases in experimental models of renal pathophysiology and human kidney^23, 24^. Eukaryotic translation initiation factor (eIF2α) phosphorylation and spliced XBP1 (sXBP1) expression were decreased in LPS-treated pod-Shp2 KO mice compared with controls indicative of decreased ER stress (Fig. 4c). We further investigated phosphorylation of Shp2 Tyr542 (a marker of enzymatic activity) and nephrin under basal and LPS-treated states. LPS induced a significant increase in Shp2 and nephrin tyrosine phosphorylation in control mice, but these were significantly reduced in pod-Shp2 KO mice (Fig. 5a,b). Collectively, these findings are in keeping with the attenuated renal and systemic inflammatory markers as well as podocyte injury in LPS-treated knockout mice.

### Shp2 deficiency in E11 podocytes attenuates LPS-induced inflammatory response

**Figure 6:**
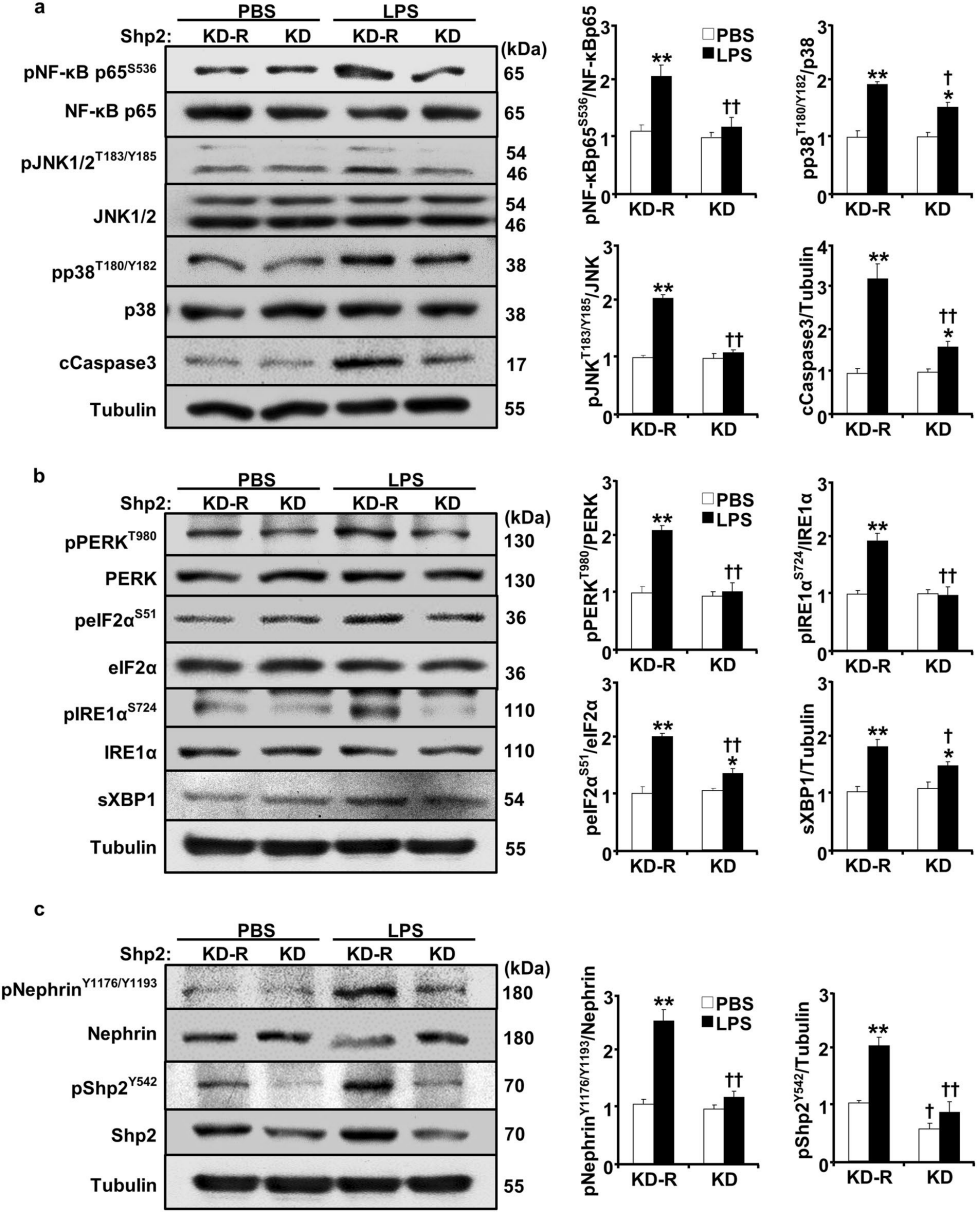
Shp2 deficiency in E11 podocytes attenuates LPS-induced inflammatory response and ER stress. Differentiated podocytes with Shp2 knockdown (KD) and rescue (KD-R) were treated with PBS and with LPS for 24 hours. Cell lysates were subjected to immunoblots with primary antibodies as indicated. Representative images are shown. Bar charts of pNF-κB p65/NF-κB p65, pJNK/JNK, pp38/p38, cleaved Caspase3/tubulin (**a**), pPERK/PERK, peIF2α/eIF2α, pIRE1α/IRE1α and sXBP1/tubulin (**b**), pNephrin/Nephrin and pShp2/tubulin (**c**) are presented as means + SEM from three independent experiments. *p < 0.05 and **p < 0.01 indicate a significant difference between PBS and LPS treatments; ^†^ *p* < 0.05 and ^††^ *p* < 0.01 indicate a significant difference between KD-R and KD podocytes. A.U., arbitrary units.

The renal inflammatory response to LPS-induced injury encompasses many cells including proximal tubules and glomerular cells (podocytes, macrophages, endothelia and mesangial). Since podocytes contribute to LPS-induced response^25^ we generated E11 podocyte cell lines with Shp2 knockdown (KD) and rescue (KD-R) to delineate the contribution of Shp2 in podocytes to LPS-induced response. Differentiated KD and KD-R podocytes were stimulated with LPS, and the effects of Shp2 deficiency on LPS-induced inflammatory response were determined. Consistent with findings in kidneys, LPS treatment increased NF-κB, JNK and p38 phosphorylation and Caspase3 cleavage and these were significantly lower in Shp2 knockdown than rescued podocytes (Fig. 6a). Moreover, Shp2 knockdown podocytes exhibited attenuated LPS-induced ER stress as evidenced by decreased phosphorylation of pancreatic ER kinase (PERK), eIF2α, inositol-requiring enzyme 1α (IRE1α) and lower sXBP1 expression (Fig. 6b). Furthermore, LPS-induced tyrosine phosphorylation of Shp2 and nephrin were significantly lower in Shp2 knockdown compared with rescued podocytes (Fig. 6c). These findings establish that podocytes contribute to the protective effects of Shp2 deficiency against LPS-induced injury.

### Shp2 deficiency reduces LPS-induced podocyte migration

**Figure 7:**
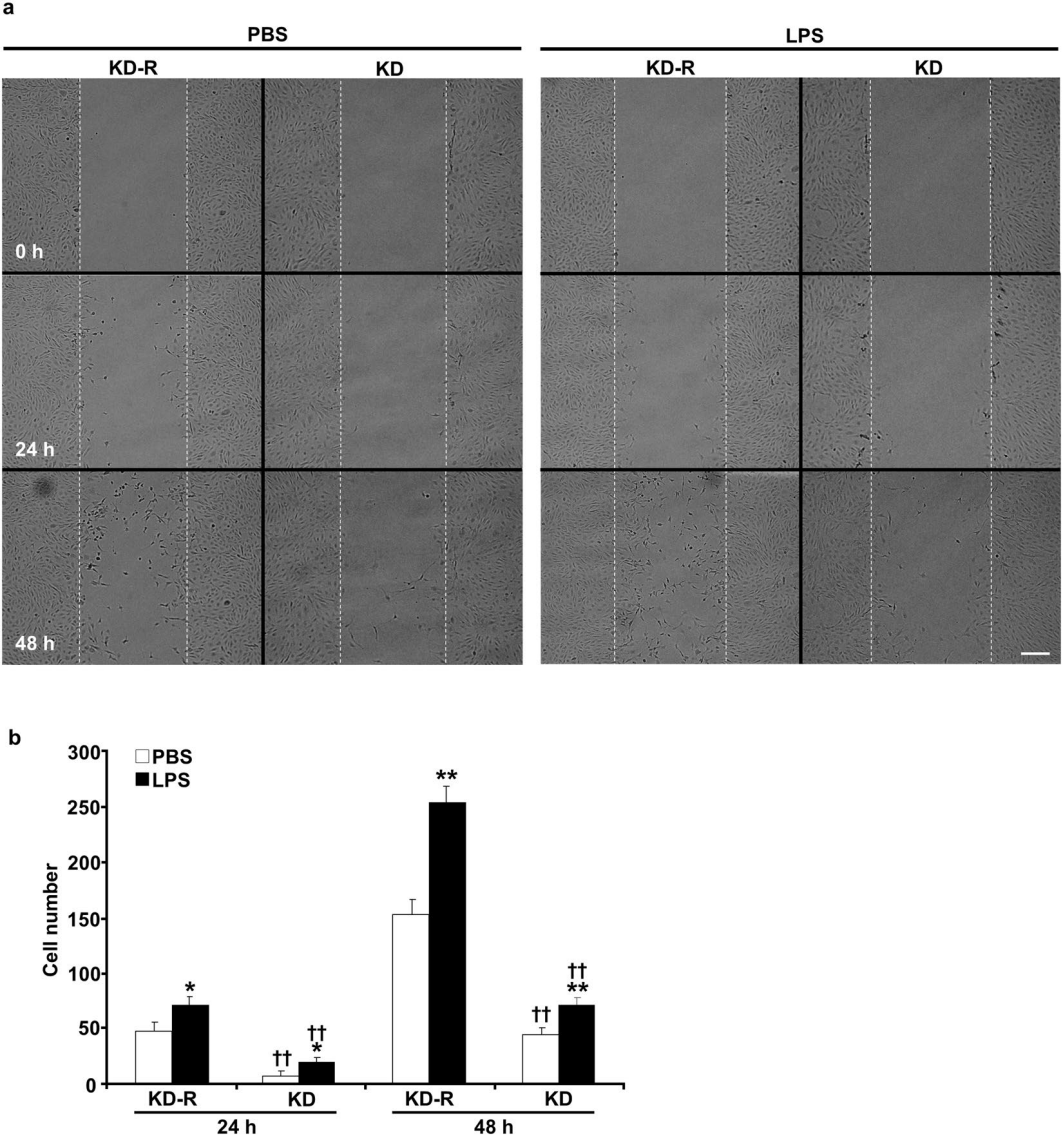
Decreased LPS-induced cell migration in Shp2 deficient podocytes. (**a**) Differentiated E11 podocytes with Shp2 knockdown (KD) and rescue (KD-R) were cultured with PBS and LPS for 48 hours after wound induction. Images were acquired before treatment (0 h) and at 24 and 48 h post wound. Scale bar: 200 µm. Cell numbers in the wound track were counted and presented in the bar chart (**b**) as means + SEM from four independent experiments. *p < 0.05 and **p < 0.01 indicate a significant difference between PBS and LPS treatments; ^††^ *p* < 0.01 indicates a significant difference between KD-R and KD podocytes.

Podocytes are terminally differentiated cells with low migration ability at physiological state. LPS-induced podocyte motility has been implicated in the pathophysiology of renal injury^26^. In addition, Shp2 plays a role in the regulation of cell migration^27–30^. Accordingly, we determined the effects of Shp2 deficiency on LPS-induced podocyte migration using wound healing assay as detailed in methods. At basal state (saline-treated) Shp2 knockdown podocytes exhibited less motility than rescued cells (Fig. 7a,b). Also, LPS treatment further enhanced podocyte migration; however Shp2 knockdown significantly decreased the number of podocytes migrating into the wound (Fig. 7a,b). These findings demonstrate that Shp2 deficient podocytes exhibit lower migration in response to LPS and are consistent with the protective effects of Shp2 podocyte deficiency *in vivo*.

## Discussion

Podocyte injury and dysfunction are significant contributors to the pathogenesis of proteinuria and glomerulosclerosis. Thus, deciphering the intricate molecular and signaling mechanisms that are implicated in podocyte dysfunction is paramount for developing therapeutic modalities for proteinuria. In the current study, we investigated the contribution of Shp2 in podocytes to LPS-induced injury. We report increased Shp2 expression in murine kidneys and podocytes following an LPS challenge. Importantly, podocyte-specific Shp2 ablation attenuated LPS-induced renal dysfunction. The protective effects of podocyte Shp2 deficiency were associated with decreased renal and systemic inflammatory response *in vivo* and were recapitulated in Shp2 deficient murine E11 podocytes. Together, these findings identify Shp2 in podocytes as a significant contributor to the signaling events following LPS challenge.

Shp2 expression increased in murine kidneys and cultured podocytes following LPS treatment. The mechanism underlying upregulation in Shp2 expression and if it translates to increased enzyme activity remain to be elucidated. However, these findings are in line with increased Shp2 expression and activity in states that are associated with elevated inflammation such as insulin resistance and high-fat diet-induced obesity^31, 32^. Also, Shp2 expression is increased in murine podocytes after puromycin-induced injury^33^. Moreover, Verma *et al*. report increased Shp2 tyrosine phosphorylation, and possibly activity, in mouse kidneys after protamine sulfate-induced injury and in a subset of human glomerular diseases (minimal change nephrosis and membranous nephropathy but not in focal segmental glomerulosclerosis)^14^. Shp2 activity was indirectly assessed through Tyr542 phosphorylation (a putative marker of increased activity since phosphorylation at this residue relieves basal inhibition)^14, 34^. In line with these findings, we observed increased Shp2 Tyr542 phosphorylation in kidneys and podocytes upon LPS challenge. It is worth noting that increased Shp2 activity during glomerular disease pathogenesis may be correlative and does not necessarily demonstrate causation. However, if causation is established then Shp2 podocyte inhibition may be of potential value in combating podocytopathies.

Shp2 ablation in podocytes attenuated LPS-induced renal dysfunction as evidenced by decreased proteinuria and blood urea nitrogen, two biomarkers of renal function. In addition, the protective effects of podocyte Shp2 deficiency were comparable in male and female mice indicating that they were not gender specific. Moreover, these effects were also observed using the nephrotoxic nephritis model demonstrating that they were not unique to a particular challenge. In this experimental platform Shp2 deficiency likely did not impact normal podocyte development due to delayed Shp2 deletion after podocyte maturation (when podocin-driven Cre recombinase is expressed^35^). Notably, the protective effects of Shp2 deletion *in vivo* were recapitulated in murine E11 podocytes with lentiviral-mediated Shp2 knockdown consistent with being cell autonomous. However, we cannot rule out direct or indirect effects of podocyte Shp2 deletion *in vivo* on other cell types/tissues that are implicated in LPS inflammatory response. Nevertheless, the current findings are consistent with the protective effects of podocyte Shp2 deficiency and pharmacological inhibition *in vivo* against protamine sulfate or nephrotoxic serum^14^. In total, the aforementioned studies establish that podocyte Shp2 deficiency and pharmacological inhibition attenuate renal injury after an acute challenge. It would be of considerable interest to further delineate the potential contribution of Shp2 in podocytes, if any, to chronic kidney diseases.

Podocyte Shp2 deficiency *in vivo* and culture attenuated LPS-induced NF-κB inflammatory response and MAPK signaling. A growing body of evidence implicates activation of NF-κB^36^ and MAPK^37^ pathways in renal inflammation and injury. Activated NF-κB in podocytes induces pro-inflammatory chemokines and aggravates proteinuria in experimental glomerulonephritis in mice^38^. Therefore, the protective effects of podocyte Shp2 deficiency against LPS may be mediated, at least partly, by decreased activation of NF-κB and MAPK pathways. The mechanism by which Shp2 deficiency attenuates MAPK and NF-κB signaling is unclear but may be related to overall reduction in inflammation. Of note, the NF-κB essential modulator NEMO regulates proinflammatory signaling through NF-κB in podocytes^39^. In addition, NEMO modulates ERK activation to enhance podocyte migration in an NF-κB independent manner^40^. Shp2 is also required for normal activation of ERK in most RTK signaling pathways, and it is interesting to speculate on interactions between Shp2 and NEMO cascades to regulate signaling during podocyte injury. Shp2 may serve as a positive^14, 41, 42^ or negative^43, 44^ regulator of the inflammatory response and is likely to act in a cell-type and tissue-/stimulus-dependent manner to elicit anti- and pro-inflammatory responses. Moreover, podocyte Shp2 deficiency affected other signaling pathways that are implicated in podocyte injury, in particular, ER stress. ER stress contributes to podocyte injury in rat primary podocytes^45^ and mouse immortalized podocytes^46^. Further, a recent study highlights the role of ER stress-derived proteinuria in the progression of human chronic kidney disease^3^. The attenuated ER stress in Shp2 deficient podocytes is consistent with the reported decrease in ER stress upon hepatic Shp2 deficiency^32^, and in line with the renal protective effects of Shp2 podocyte deficiency. While our findings provide insights into the signaling mechanisms that are associated with Shp2 deficiency following LPS injury they do not identify the target(s) that mediate Shp2 actions in podocytes. Shp2 associates with and enhances tyrosine phosphorylation of the slit diaphragm protein nephrin^14^, which is also regulated by Shp1^9^ and PTP1B^7, 8^ in podocytes. In line with these observations, we demonstrated increased nephrin phosphorylation concomitant with elevated Shp2 tyrosine phosphorylation upon LPS challenge, suggesting that nephrin may be a component of the signaling cascade that mediates Shp2 action upon LPS-induced renal injury. Also, regulatory proteins for cell-matrix interactions such as focal adhesion kinase and urokinase receptor promote cell migration, and their podocyte deficiency protects against LPS-induced proteinuria^26, 47^. Shp2 activity positively regulates the activation of focal adhesion and integrin complex^29, 48–50^. Consistent with these observations, Shp2 deficient E11 podocytes exhibited decreased migration in wound healing after LPS challenge. Additional studies are needed to delineate the molecular signaling mechanisms mediating Shp2 actions in podocytes, but the current findings suggest that Shp2 inhibition in podocytes may be of potential therapeutic value for podocytopathies.

As an essential regulator of various RTK signaling pathways, Shp2 was reported as oncogenic tyrosine phosphatase^51^. Also activating Shp2 mutants have been studied in association with developmental disorders such as Noonan syndrome^52^ and are found in several human cancers^53^. Accordingly, several Shp2 pharmacological inhibitors are being developed for cancer therapy^54^ including a promising allosteric inhibitor that exhibits high specificity and capacity to suppress proliferation in RTK-driven human cancer cells and mouse tumor xenograft models^55^. Previous^14^ and current studies establish protective effects of podocyte Shp2 deficiency against acute renal injury raising the possibility of utilizing Shp2 pharmacological inhibitors to combat podocytopathies.

## Materials and Methods

### Mouse studies

Shp2 floxed (Shp2^fl/fl^) mice on a mixed 129Sv/J × C57Bl/6J background were generated and described previously^56^. Transgenic mice expressing Cre recombinase under control of podocin-specific promoter NPHS2 on the C57Bl/6J background were purchased from Jackson laboratories. Shp2^fl/fl^ were bred to podocin-Cre to generate mice lacking Shp2 in podocytes (pod-Shp2 KO). DNA extracted from tails was used for genotyping of the Shp2 floxed allele and for the presence of Cre by polymerase chain reaction (PCR). Primers used to check for germline deletion were: forward 5′-TAGCTGCTTTAACCCTCTGTGT and reverse 5′-AATTGCGGCTTCTTGTCCT. All mice studied were age-matched and were maintained on a 12 h light-dark cycle, with free access to water and standard laboratory chow (Purina lab chow, # 5001). For lipopolysaccharide (LPS)-induced proteinuria, 10–12 weeks old control and pod-Shp2 knockout mice received a single intraperitoneal injection of LPS (Sigma, from *Escherichia coli* 0111:B4; 16 µg/g body weight) in 0.9% sterile saline solution^17^. Mice were sacrificed 24 h after injection of LPS and kidneys were harvested and processed for biochemical and histological analyses. Urine and blood samples were collected one hour before and at sacrifice, respectively. For the nephrotoxic nephritis model, 10–12 weeks old control and pod-Shp2 KO mice were injected retro-orbitally with sheep anti-rat glomerular serum (Probetex, 5 µl/g body weight) or saline. Urine samples were collected at 24 and 48 h, and blood samples were collected at 48 h post injection. All the mouse studies were conducted according to federal guidelines and were approved by the Institutional Animal Care and Use Committee at the University of California-Davis.

### Metabolic measurements

Urinary protein was determined by Bradford method using Protein Assay Dye Reagent Concentrate solution (Bio-Rad). Albumin and creatinine concentrations were measured using corresponding kits that were purchased from Sigma. Blood plasma samples were collected in EDTA-treated tubes by centrifugation at 2,000× g for 20 minutes to deplete platelets, and inflammation-related biomarkers were assayed using the V-PLEX kit (Meso Scale Discovery). In addition, blood urea nitrogen was measured using urea assay kit (Sigma). All procedures were performed according to manufacturer instructions.

### Immunohistochemistry

Kidney samples from control and pod-Shp2 knockout mice were fixed in 4% paraformaldehyde, embedded in paraffin and deparaffinized in xylene. Sections were stained using synaptopodin, Shp2 (Santa Cruz) and pNephrin (Tyr1176/Tyr1193) antibodies at 4 °C overnight. Detection was performed with appropriate Alexa Fluor-conjugated secondary antibodies (Thermo Fisher Scientific) and then visualized using Olympus BX51 microscope (Fig. 2c) or Olympus FV1000 Laser Scanning Confocal microscope (Figs 1b and 5b).

### Podocyte isolation

Podocytes were isolated from control and pod-Shp2 knockout mice using established protocols^57, 58^ with some modifications. Briefly, kidneys were decapsulated and minced in Krebs-Henseleit saline (119 mM NaCl, 4.7 mM KCl, 1.9 mM CaCl_2_, 1.2 mM KH_2_PO_4_, 1.2 mM MgSO_4_ and 25 mM NaHCO_3_, pH 7.4). Samples were pooled and filtered through sieves in order of 250, 100 and 40 µm. The fraction trapped on 40 µm sieve containing glomeruli was washed and collected, then digested in Hanks buffer supplemented with 0.1% collagenase D (Roche) and 0.25% trypsin (Gibco) for 30 min at 37 °C. Solutions were filtered through a 40 µm sieve and podocytes centrifuged at 1500 × g for 5 minutes. Cell pellets were lysed using radio-immunoprecipitation assay (RIPA) buffer containing 20 mM Tris-HCl (pH 7.4), 150 mM NaCl, 0.1% SDS, 5 mM EDTA, 20 mM NaF, 10 mM Na_4_P_2_O_7_, 1% Triton X-100, 1% sodium deoxycholate and protease inhibitors.

### Cell culture

E11 murine kidney podocyte cell line was purchased from Cell Lines Service (Eppelheim, Germany). E11 podocytes are proliferative when maintained at 33 °C in RPMI 1640 medium supplemented with 10% fetal bovine serum (FBS). To induce differentiation, E11 cells were cultured at 37 °C for two weeks. Shp2 silencing was achieved by testing five individual hairpin shRNAs (GE Healthcare Dharmacon) through lentiviral infection. Lentiviruses were produced by co-transfection of packaging (psPAX2) and envelope (pMD2.G) vectors (Addgene) in HEK293FT cells using Lipofectamine 2000 (Invitrogen) following the manufacturer’s guidelines. Infected E11 podocytes were selected by puromycin (2 µg/ml) to generate stable colonies. E11 cells with Shp2 knockdown (KD) were rescued (KD-R) with Shp2 by transfection with pCMV human Shp2 wild type (Addgene), and stable cell lines were generated by neomycin (G418, 400 µg/ml) selection. For biochemical studies differentiated KD and KD-R podocytes were starved for 24 h then stimulated with LPS (10 μg/ml) for indicated times. For wound healing assays differentiated KD and KD-R podocytes were starved for 24 h then wound was generated using 200 μl pipette tip. Cells were incubated in RPMI medium (10% FBS) plus LPS (10 μg/ml) for 48 h and images acquired using Olympus BX51 microscope at indicated times. Quantitation of cell migration was performed by manual counting of cells in the wound using enlarged images.

### Biochemical analyses

Tissues and cells were lysed in RIPA buffer. Lysates were centrifuged at 18,000 × g for 10 min and protein concentrations determined by bicinchoninic acid protein assay kit (Pierce Chemical). Proteins (30 μg for cell lysates and 60 μg for tissue lysates) were resolved by SDS-PAGE and transferred to PVDF membranes. Immunoblotting was performed as we previously described^59, 60^ using antibodies for pNF-κBp65 (Ser536), NF-κBp65, pp38 (Thr180/Tyr182), p38, pJNK (Thr183/Tyr185), JNK, pShp2 (Tyr542) (all from Cell Signaling), Shp2, pPERK (Thr980), PERK, peIF2α (Ser52), eIF2α, sXBP1, IRE1α, Nephrin, actin and tubulin (all from Santa Cruz). Antibodies for pIRE1α (Ser724) and pNephrin (Tyr1176/Tyr1193) were purchased from Abcam. Proteins were visualized using HyGLO chemiluminescence HRP detection reagent (Denville Scientific), and pixel intensities of immuno-reactive bands were quantitated using FluorChem 9900 software (Alpha Innotech). Data for phosphorylated proteins were presented as phosphorylation level normalized to protein expression.

Total RNA was extracted from kidneys using TRIzol (Invitrogen) and cDNA generated using high-capacity cDNA Synthesis Kit (Applied Biosystems). mRNA levels were assessed using SYBR Green quantitative real-time PCR using SsoAdvanced Universal SYBR Green Supermix (iCycler, Bio-Rad). Relative gene expression was normalized to Tata-box binding protein (*Tbp*) and quantitated using the 2^−ΔΔ*C*^_T_ method as we previously described^61^. Primers sequences are listed in Table 1.

**Table 1:** List of primers used to determine mRNA of Shp2 and pro-inflammatory cytokines.

Gene	Forward 5′->3′	Reverse 5′->3′
PTPN11	AGTTACATTGCCACTCAAGGCTGC	TTGACGTTCCTAACACGCATGACC
Il-1b	AGCTTCAGGCAGGCAGTATC	AAGGTCCACGGGAAAGACAC
Il-6	ACAACCACGGCCTTCCCTACTT	CACGATTTCCCAGAGAACATGTG
Tbp	TTGGCTAGGTTTCTGCGGTC	GCCCTGAGCATAAGGTGGAA
Tnfa	GACGTGGAACTGGCAGAAGAG	TGCCACAAGCAGGAATGAGA

### Statistical analyses

Data were expressed as means + standard error of the mean (SEM). Statistical analyses were performed using an unpaired heteroscedastic two-tailed Student’s *t*-test for Fig. 1a and one-way ANOVA tests for the other experiments. Differences were considered significant at *p* < 0.05.

## Electronic supplementary material

Supplementary Information
