# Supplementary material for: Protein tyrosine phosphatase Shp2 deficiency in podocytes attenuates lipopolysaccharide-induced proteinuria
Source: Sci Rep. 2017 Mar 28;7:461. doi: 10.1038/s41598-017-00564-3 (PMC5428720; doi:10.1038/s41598-017-00564-3)
Supplement: Supplementary file 1 — Supplementary Information [file 41598_2017_564_MOESM1_ESM.pdf]

**Supplementary Information**

# Protein tyrosine phosphatase Shp2 deficiency in podocytes attenuates lipopolysaccharide-induced proteinuria

Ming-Fo Hsu[1], Ahmed Bettaieb[1†], Yoshihiro Ito[1], James Graham[1,2], Peter J. Havel[1,2], Fawaz G. Haj[1,3,4*]

[1] Department of Nutrition, University of California Davis, One Shields Ave, Davis, CA 95616

[2] Department of Molecular Biosciences, School of Veterinary Medicine, University of California Davis, One Shields Ave, Davis, CA 95616

[3] Comprehensive Cancer Center, University of California Davis, Sacramento, CA 95817

[4] Division of Endocrinology, Diabetes and Metabolism, Department of Internal Medicine, University of California Davis, Sacramento, CA 95817

[*] Corresponding author: Fawaz Haj, D.Phil. University of California Davis, Department of Nutrition, 3135 Meyer Hall, Davis, CA 95616, Tel: (530) 752-3214, e-mail: fghaj@ucdavis.edu

[†] Current address: Department of Nutrition, University of Tennessee-Knoxville, Knoxville, TN 37996

Running title: Podocyte Shp2 deficiency and renal injury

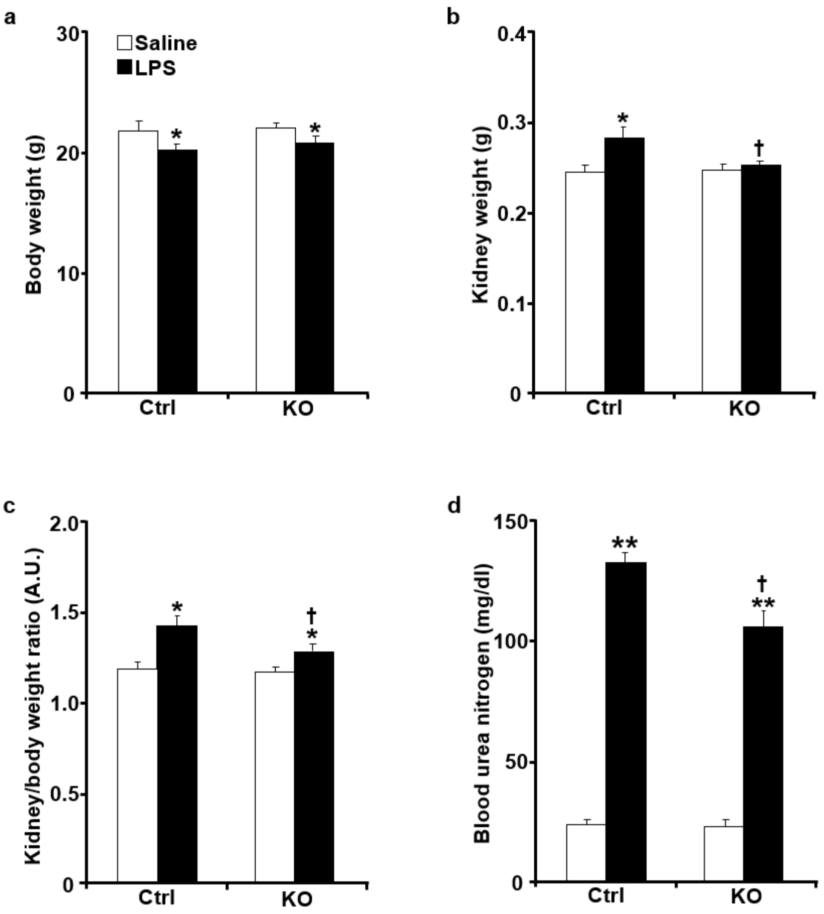


**Supplementary Figure S1: Pod-Shp2 KO female mice are more resistant than controls to LPS-induced renal injury.** Changes in body weight (**a**), kidney weight (**b**), kidney/body weight ratio (**c**) and blood urea nitrogen (**d**) of female control (Ctrl, n=7) and pod-Shp2 knockout (KO, n=8) mice without (saline) and with LPS treatments. *$p$<0.05 and **$p$<0.01 indicate significant difference between saline and LPS treatments; $^{\dagger}p$<0.05 indicates significant difference between Ctrl and KO mice. Data were presented as means ± SEM. A.U., arbitrary units.

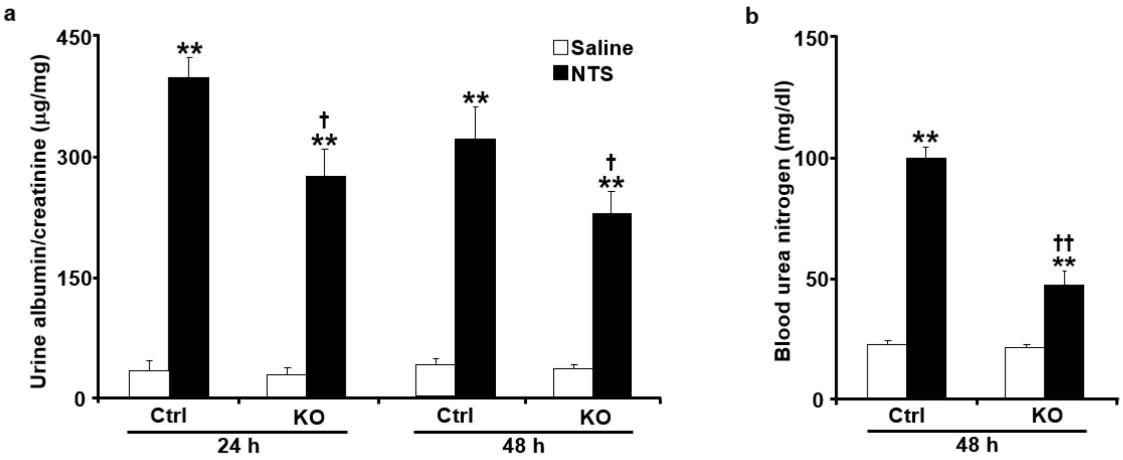


**Supplementary Figure S2: Pod-Shp2 KO mice are more resistant than controls to the nephrotoxic nephritis model of renal injury.** Changes in urine albumin/creatinine ratio (**a**) and blood urea nitrogen (**b**) of female control and pod-Shp2 knockout mice without (saline, n=8 for each group) and with NTS (n=10 for each group) treatments. **$p$<0.01 indicates significant difference between saline and NTS treatments; $^{\dagger}p$<0.05 and $^{\dagger\dagger}p$<0.01 indicate significant difference between Ctrl and KO mice. Data were presented as means $\pm$ SEM.

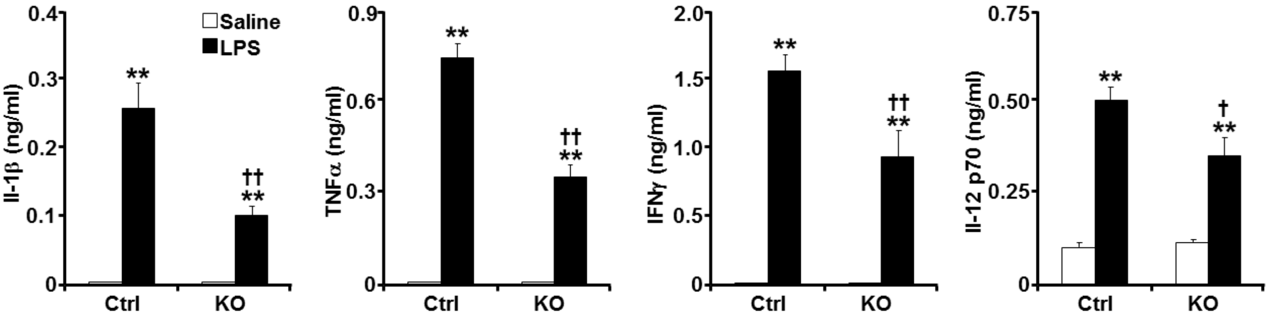


**Supplementary Figure S3: Attenuated LPS-induced inflammatory response in pod-Shp2 KO female mice.** Plasma concentrations of IL-1β, TNFα, INFγ and IL-12 p70 in saline and LPS-treated female control (n=6) and pod-Shp2 KO (n=6) mice. $^{**}p<0.01$ indicates significant difference between saline and LPS treatments; $^{\dagger}p<0.05$ and $^{\dagger\dagger}p<0.01$ indicate significant difference between Ctrl and KO mice.
